# Differences in medication reconciliation interventions between six hospitals: a mixed method study

**DOI:** 10.1186/s12913-022-08118-8

**Published:** 2022-05-31

**Authors:** C. C. M. Stuijt, B. J. F. van den Bemt, V. E. Boerlage, M. J. A. Janssen, K. Taxis, F. Karapinar-Çarkit

**Affiliations:** 1 ApoMed, Michelangelostraat 62-2, 1077CG Amsterdam, the Netherlands; 2 Department of Pharmacy, Sint Maartenskliniek, Ubbergen, the Netherlands; 3 Department of Pharmacy, Radboud University Medical Center, Nijmegen, the Netherlands; 4 Department of Pharmacotherapy and Pharmaceutical Care, University of Groningen, Groningen, the Netherlands; 5 Department of Clinical Pharmacy, OLVG, Amsterdam, the Netherlands

**Keywords:** Medication reconciliation, Medication errors, Hospital admission, Hospital discharge, Hospital pharmacy

## Abstract

**Background:**

Although medication reconciliation (MedRec) is mandated and effective in decreasing preventable medication errors during transition of care, hospitals implement MedRec differently.

**Objective:**

Quantitatively compare the number and type of MedRec interventions between hospitals upon admission and discharge, followed by a qualitative analysis on potential reasons for differences.

**Methods:**

This explanatory retrospective mixed-method study consisted of a quantitative and a qualitative part. Patients from six hospitals and six different wards i.e. orthopaedics, surgery, pulmonary diseases, internal medicine, cardiology and gastroenterology were included. At these wards, MedRec was implemented both on hospital admission and discharge. The number of pharmacy interventions was collected and classified in two subcategories. First, the number of interventions to resolve unintended discrepancies (elimination of differences between listed medication and the patient’s actual medication use). And second, the number of medication optimizations (optimization of pharmacotherapy e.g. eliminating double medication). Based on these quantitative results and interviews, a focus group was performed to give insight in local MedRec processes to address differences in context between hospitals. Descriptive analysis (quantitative) and content analysis (qualitative) was used.

**Results:**

On admission 765 (85%) patients from six hospitals, received MedRec by trained nurses, pharmacy technicians, pharmaceutical consultants or pharmacists. Of those, 36–95% (mean per patient 2.2 (SD ± 2.4)) had at least one discrepancy. Upon discharge, these numbers were among 632 (70%) of patients, 5–28% (mean per patient 0.7 (SD 1.2)). Optimizations in pharmacotherapy were implemented for 2% (0.4–3.7 interventions per patient upon admission) to 95% (0.1–1.7 interventions per patient upon discharge) of patients. The main themes explaining differences in numbers of interventions were patient-mix, the type of healthcare professionals involved, where and when patient interviews for MedRec were performed and finally, embedding and extent of medication optimization.

**Conclusions:**

Hospitals differed greatly in the number of interventions performed during MedRec. Differences in execution of MedRec and local context determines the number of interventions. This study can support hospitals who want to optimize MedRec processes.

**Supplementary Information:**

The online version contains supplementary material available at 10.1186/s12913-022-08118-8.

## Introduction

Medication errors upon hospital admission and discharge are common and can lead to preventable adverse drug events (ADEs) [1, 2]. To diminish these errors, medication reconciliation(MedRec) is recommended in many countries: several studies have demonstrated substantial reduction in medication errors, specifically medication discrepancies, and, to some extent, in ADEs [3–7].

In fact, MedRec consists of three different steps which are described by the Healthcare Improvement Institute (USA): 1. verification (eliminating discrepancies between a patients’ actual medication use and in-hospital prescriptions by comparing medication overviews and interviewing patients), 2. clarification (medication optimization, e.g. start of a laxative if an opioid is prescribed, eliminating double medications) and 3. reconciliation (discussion of step 1 and 2 and reasons for medication changes with the physician and documentation of this information) (Additional file 1: box 1a) [6].

Despite this clear definition, there is remarkable diversity in reported effectiveness of MedRec. For example, in the verification step detected discrepancies between the actual medication use of a patient and the medication list constituted in the hospital, was found to vary between 3.4 and 98% [8–11]. This broad range may be explained by variances in study methodology, differences in study population (e.g. acute admissions, elderly and high numbers of admission medication), staff available for MedRec but also in dissimilarities in definition [12]. The latter may result in a variety of interpretations or implementation of the distinct steps of MedRec [11, 13–15]. This will give direction to optimize MedRec processes in hospitals which is highly needed due to shortage in resources. Furthermore, the clarification step is not implemented frequently: it may be included in verification- and reconciliation steps without explicit reporting or not at all being executed [16, 17]. Knowledge on inter-hospital variability of MedRec processes upon hospital admission and discharge, may give insight in MedRec-strategies and the impact on numbers of interventions. This will give direction to optimize MedRec processes in hospitals which is highly needed due to shortage in resources [11, 13].

Current literature describes the impact of available staff, the employee who performs MedRec, hospital stay duration and number of admission medications on the number of interventions. However, no study performed a broader, in depth analysis to compare MedRec processes between hospitals in a real-world setting [14]. Hence, the objective of this mixed-method study is to quantitatively compare the number and type of MedRec interventions between hospitals upon admission and discharge, followed by a qualitative analysis of potential reasons for these differences.

## Methods

### Study design

An explanatory mixed method study was performed, consisting of a quantitative and a qualitative part.

### Ethics

The Institutional Review Board, The Nijmegen ethics committee, Concernstaf Kwaliteit en Veiligheid—Commissie Mensgebonden Onderzoek, University Medical Center Radboud, reviewed the study and confirmed compliance with the Dutch legislation by giving the waiver of approval (registration nr 2013/328**).**

### Quantitative part

#### Setting

A retrospective cohort study was performed. Hospitals were selected if they executed MedRec for at least five years, both upon hospital admission and discharge. As this study focused on MedRec in a real-world setting, included wards varied based on the MedRec activities of each hospital (orthopaedics, surgery, pulmonary diseases, internal medicine cardiology and gastroenterology; Table 1).

**Table 1:** hospital and overall included patient characteristics

*Hospital*	A	B	C	D	E	F
*County (region)*	Capital (West)	Drenthe (North)	Gelderland (East)	Zuid Holland (West)	Utrecht (Centre)	Limburg (South)
*surrounding*	Urban	Rural	Rural	Urban	Urban	Urban
*Type*	Teaching	General	Specialized	General	Teaching	Teaching
*Number of beds 2012*	551	284	317	722	1102	536
*Wards with MR activities by pharmacies in 2012*	Lung diseases Internal medicine Cardiology Neurology	All wards including ICU, paediatrics	All wards	75% all admissions covered; discharge counselling if patient passes by outpatient pharmacy or on request physician	All wards except ICUs	All wards except ED
*Study wards and number of patients included (n)*	Lung diseases (150)	Internal medicines (27) Cardiology (50) Surgery (73)	Orthopaedics (150)	Internal medicines (23) Gastroenterology (4) Surgery (114) Lung diseases (8)	Internal medicines (107) Gastroenterology (12) Surgery (28) Lung diseases (3)	Internal medicines (85) Lung disease (65)
*length of stay, median (range)*	9.0 (3–48)	4.0 (1–60)	6.0 (1–115)	6.0 (1–21)	7.0 (2–32)	7.0 (2–37)
*number admission medications, mean (SD)*	9.1 (4.5)	6.4 (3.4)	-^a^	8.5 (4.1)	10.8 (4.3)	7.8 (4.3)
*number discharge medications, mean (SD)*	10.9 (4.8)	8.2 (4.0)	11.1 (4.1)	8.8 (3.6)	10.4 (4.3)	-
*Number admission/discharge interviews*^b^	108/149	140/92	145/136	147/149	78/106	146/-
*age,mean (SD)*	67.2 (13.3)	62.1 (16.5)	61.2 (12.9)	69.3 (12.4)	66.1 (14.6)	69.1 (13.0)
*female, n (%)*	44 (41)	80 (57)	97 (67)	84 (57)	46 (59)	79 (54)
*low social class, n (%)*	81 (75)	83 (59)	46 (32)	57 (39)	13 (17)	85 (58)
*deprived area, n (%)*	48 (44)	0	2 (1)	10 (7)	4 (5)	4 (3)

Between hospitals: age, number of admission- and discharge medications, length of stay showed significant differences (p = 0.000)

^a^no differentiation on admission or pre-admission medication was possible

^b^Deviations from number of included patients: Admission: 134 patients had no interview, 69 (51%) patients of whom were admitted to hospital E and met their exclusion criteria for medication reconciliation, 44 (25%) were incapable of being interviewed without the presence of a caregiver or had a language barrier and 21 (16%) had the MedRec interview upon being discharged due to a short length of stay

Upon discharge, 632 (70%) patients were interviewed and included. Patients from hospital F (*n* = 150, 16%) were excluded due to incomplete documentation regarding intervention performance, 117 patients (15%) were missed due to an unexpected discharge

#### Study population

All consecutive patients admitted to one of the six selected hospitals were included if a patient had medication intended for chronic use before admission. Patients received MedRec both upon hospital admission and discharge. All included patients had had at least a discharge interview, those without an admission interview (e.g. due to a short length of stay) were included in case the interview could be executed upon discharge. Patients incapable to be interviewed e.g. with a language barrier were excluded. Also, patients living in an institutionalised setting before admission were excluded (presuming dependence in medication administration with consequent inability of assessing a medication history from the patient or their proxy). Per hospital we included 150 patients (900 total) based on a previous analysis on the same data [11].

#### Medication reconciliation

At the time of inclusion most patients (> 90%) attended one community pharmacy (CP) [18]. Here, prescriptions from multiple prescribers are documented. Therefore, a medication history from the CP combined with a patient interview is considered the gold standard to obtain the Best Possible Medication History (BPMH) in the Netherlands [19, 20]. In case of doubt the GP could be consulted. Generally, the medication history of the CP is electronically available, otherwise it was obtained by fax [16, 21].

MedRec is fulfilled as described in Additional file 1: box 1a and performed by (specialized) pharmacy technicians with background support of pharmacists. Pharmacy technicians have shown to perform MedRec accurately in the Netherlands [22]. They have had a three year intermediate vocational training, which involves a combination of study at a college or open learning, in addition to practical working experience. A pharmacy technician can specialise further into pharmaceutical consultant, who have received an additional 3 year bachelor training focused on pharmacotherapy and communication.

#### Quantitative outcomes

Number and type of interventions per patient in the verification - and clarification step of MedRec, i.e. resolving unintentional medication discrepancies and optimizations of pharmacotherapy upon admission and discharge.

#### Data collection

All data were collected by three trained data collectors from hospital patient records and admission/discharge pharmacy checklists. Participating pharmacists and pharmacy technicians documented proposed medication changes (interventions) on their checklist, communicated with the physician in charge of the patient, who would follow or reject the advice. Accepted interventions (omission of chronic medication e.g. metoprolol) were counted, non-accepted interventions were regarded as either an intentional medication change (e.g. omission of frusemide for a patient who was admitted with dehydration) or a non-relevant suggestion for an intervention (e.g. multivitamin use at home). In case interventions were not clearly documented, pharmacy teams were available to clarify or, if no explanation could be provided, the intervention was discarded.

Each accepted discrepancy or optimization was counted as an unique intervention. A single drug could therefore induce several interventions, e.g. restarting furosemide on admission to correct a discrepancy and adjusting the dose upon discharge back to the dose used at home after a temporal dose increase (this was counted as two interventions, one upon hospital admission and one upon discharge). In case a discrepancy between the CP list and patient reported use of medicines was noticed (including potentially stopped medication during admission), this was checked in the electronic medical record and documented as stated by the patient with discussion remarks. In case of doubt about the correct recall on medicines use of the patient, additional measures were implemented (e.g. asking for medication boxes, contacting GP).

Hospitals defined *unintended medication discrepancies* as differences among medication regimens i.e., between actual use of a patient’s home regimen and medications prescribed upon admission or discharge [23].

*Optimizations of medication* entail a check on whether the medication list is adequate and optimal regarding (high risk) medication (e.g. NSAID use in combination with gastro-protection in elderly, laxative with opioid use based on guidelines), on duplication of therapy and on discontinuation of temporarily indicated medication (e.g. discontinuation opioids and laxatives, hypnotics, proton pump inhibitors initiated during admission but without indication upon hospital discharge. Both discrepancies and optimizations were recorded as described by Karapinar et al. and classified into the categories as described in Additional file 1: box 1b: *start*, *dosage*, *switch* and *discontinuation/stop *[16].

The following covariates were collected: number of chronic drugs (based on BPMH upon hospital discharge), demographic data including age, gender and socioeconomic status (via postal-code) [24, 25]. Postal-codes were also used for deprived neighbourhoods as registered by The Dutch Healthcare Authority (NZA) [26].

#### Analysis

Statistics were executed with SPSS 23. Total and mean numbers of interventions both on medication discrepancies and optimizations per patient were calculated in patients with either an admission-, or discharge interview. Also, the proportion of patients with at least one intervention on an unintended discrepancy and medication optimization was assessed. Mean (standard deviation) or median (range) were determined dependent on the distribution of data. Discharge data of hospital F had to be excluded due to the high number of unknown acceptance rate of interventions.

Ideally, a multi-regression model (e.g. multilevel analysis) should have been used in order to perform a proper comparison among hospitals. However, we noted several unmeasured confounding factors (e.g. health literacy of patients in rural versus urban areas, differences in definitions for MedRec, different degrees of patient involvement and different levels of experience of healthcare professionals performing MedRec), which made it impossible to compare hospitals in a robust way. Therefore, we decided to address these context differences between hospitals as described in the qualitative part.

To support the discussion for the qualitative part, hospitals were compared, regarding their case-mix, normally distributed continuous variables including age, number of admission- and discharge medications was analysed by ANOVA. For analysis on differences in gender, socioeconomic status, deprived area, length of hospital stay, ward and admission type, the Pearson Chi-square test was used.

### Qualitative part

#### Design

A qualitative explorative study using individual interviews and a focus group interview, was conducted.

#### Participants

Responsible pharmacists (1 to 2 per hospital) and pharmacy technicians (2—4 per hospital) from included hospitals participated in the quantitative part of the study and were present at the time of investigators visit for individual interviews. These participants were also eligible for the focus group and were selected as they were involved in MedRec, made locally choices on the implementation of MedRec and had knowledge on the process.

In this focus group, which was organised in July 2015, participated five pharmacists in person, one was contacted by phone. A representative of each hospital participated.

#### Data collection

To understand how an individual hospital had implemented MedRec an inventory questionnaire was used to get knowledge on (hospital)pharmacy components of MedRec activities and feasibility of data collection. Questions included were for instance: how was the MedRec process performed (e.g. were all 4 steps implemented, see box) and what type of staff members performed MedRec (see Table 2 for all topics). The questionnaire was completed during an in person, semi-structured interview with participants. All interviews were conducted by the first investigator (pharmacist, > 20 years’ experience in community -, outpatient- and hospital pharmacy). The questionnaire was developed based on literature, experience and expert-discussion (FK, BvB).

**Table 2:** categories and sub-classifications extracted from interview and focus group

**WHO**	**A**	**B**	**C**	**D**	**E**	**F**
Patient selection:	Non	Non	Non	Non	High risk patients^a^	Exclusion UDS^b^
Type of pharmacy team members involved:	Pharmaceutical consultant	Pharmacy technician + pharmacist check	Pharmacy technician + pharmacist check	Pharmacy technician + specialized pharmacy technician + pharmaceutical consultant	Specialized pharmacy technician + pharmaceutical consultant + pharmacist	Pharmacy technician
			Nurse	Nurse		
**WHERE (location interview)**	**A**	**B**	**C**	**D**	**E**	**F**
***Admission***						
	On the ward	Outpatient clinic (elective)	@ home (mail/phone)	Elective patients: @ home (mail/phone)	@ home (mail/phone)	Outpatient clinic (elective)
		On the ward	On the ward	On the ward	On the ward	On the ward
***Discharge***						
	On the ward	Outpatient pharmacy^d^	On the ward	Outpatient pharmacy	On the ward	Outpatient pharmacy
			Outpatient pharmacy		Outpatient pharmacy + @home by phone	
**HOW (process)**	**A**	**B**	**C**	**D**	**E**	**F**
***Admission***						
*Verification*: Information collection	digital or paper-based pharmacy dispensing information and GP	Digital pharmacy dispensing information	Patient list, extra check high risk patients^c^, lab results	Digital pharmacy dispensing information	digital or paper-based pharmacy dispensing information and GP	digital or paper-based pharmacy dispensing information
*Clarification*: Optimisation medication	8 focuspoints, generally discussed @ discharge	Thrombo-profylaxis, oral antidiabetics	see above: focus on renal function, pain meds	No extra checks	see discharge	no extra checks
	Substitution		No substitution		Substitution	
Suggested medication changes	implementation of medication changes after check with physician	implementation of medication changes by pharmacy without physician check	implementation of medication changes after check with physician	Only acutely based on prescriptions	discussion with doctor after medication review	Only acutely based on prescriptions
Documentation	On electronic dispensing information from pharmacy and checklist (not visible for other healthcare professionals)	On checklist (not visible for other healthcare professionals)	On Checklist (not visible for other healthcare professionals)	? not visible for other healthcare professionals	In EPD and on electronic dispensing information from pharmacy (partly available for other healthcare professionals)	Electronic pre-registration (elective patients) available for other healthcare professionals
***Discharge***						
Optimisation (number of focuspoints)	8	2	2	0	6^e^	3 (surgical ward only)
**WHEN (timing activity)**	**A**	**B**	**C**	**D**	**E**	**F**
***Admission***						
Interview elective patients (moment) ***Discharge***	Day admission	Day admission	Weeks before procedure by mail	Weeks before procedure	Weeks before procedure by phone	Weeks before procedure
Interview will be performed	If discharge announced 24 h beforehand	Only if patient passes by outpatient pharmacy	Always	Only if patient passes by outpatient pharmacy	If discharge announced 24 h beforehand	Only if patient passes by outpatient pharmacy

^a^Patient interview only in case of > 3 chronic drugs + 50 years or over (70–80% of all patients)

^b^patients with drugs dispensed in unified dosing systems (UDS, Baxter)) are not counselled

^c^extra check for high risk patients right before admission (age > 65 years and > 5 chronic medications). All patients fill out their own medication lists before the elective admission

^d^An *outpatient pharmacy* is a pharmacy based in the outpatient clinic of a hospital with community pharmacy activities, generally with close connections with the hospital pharmacy

^e^all issues during medication review if applicable + check on renal function and electrolytes

To ensure correct interpretation of collected information, all interviewees were sent the outcomes of the questionnaire to provide feedback on data (member check).

To understand the causes for differences in numbers of MedRec interventions between hospitals, an explanatory focus group was conducted. During this focus group, we reached a point of ‘theoretical saturation’ as focus group members could not provide new concepts regarding the differences in the quantitative results.

An independent moderator (KT) led the group and FK acted as an observer to document relevant contributions that would not necessarily have been picked up by audio-recording. Interviewees gave written informed consent for audio recording and anonymity was guaranteed.

Before the meeting, all attendees were provided with drafts of the quantitative results of all hospitals and figures to get a clear overview of processes in each centre (derived from the inventory questionnaire), patient characteristics and numbers and types of interventions performed in each hospital. KT proposed to touch upon the following themes (based on differences between hospitals in the quantitative results): differences in processes, persons performing MedRec and patient-mix.

The audio-recording was transcripted verbatim by an official transcriptor. Also, a member check was conducted by sending participants a summary of the results in order to provide feedback on the factual and interpretive accuracy of the data.

#### Qualitative Outcome

Explanations for differences in numbers of MedRec interventions by a focus group discussion.

#### Analysis

For the data analysis a content analysis was performed. Based on the focus group discussion, an inductive, content analysis was applied on the transcripts. First, relevant text fragments were selected individually by three researchers (CS, FK, BB) and compared to ensure no data would be missed. Second, the first researcher performed the open coding of the fragments, and applied axial coding. Relationships between the open codes were identified with axial coding and the codes were labelled into themes. This process was reviewed in its entirety by two researchers (FK, BB) until all researchers fully agreed on the content of the themes [27].

## Results

### Quantitive part

Overall, 899 of 900 patients were included (one exclusion due to missing information on medication use). On admission 765 (85% of 899) and upon discharge 632 (70%) patients received MedRec (Table 1).

Most participants were elderly patients with a low socioeconomic status (50%), mean age of 65 years, an equally distributed gender and were mostly admitted on internal medicines, lung diseases and surgery. Overall, acute-, and elective admissions were equally distributed. However, individual hospitals varied highly in the proportion of acute admissions (0–100%), median length of stay (4—9 days), mean number of admission medications (6.4 -10.8) and socioeconomic status (17–75% in the lowest category) (Table 1).

#### Number and types of interventions

Overall, 2309 (74%) interventions were accepted by physicians; 1675 interventions (in 765 interviewed patients) were collected on admission and 634 (632 patients) upon discharge. The mean number of accepted interventions was 2.2 (SD 2.4) per patient on admission and 0.7 (SD 1.2) upon discharge. Start and dosage interventions occurred most frequently on admission, whereas start interventions reached the highest number upon discharge.

#### Unintended discrepancies (verification)

On admission, the proportion of patients with at least one discrepancy varied between hospitals from 36 to 95%. Upon discharge, this ranged from 5 to 28% of patients.

Overall, hospitals varied with regard to the distribution of types of interventions. In general, start and dosage interventions were most frequently implemented, as medication a patient used pre-admission was omitted or patients used different dosages. Also, stops and switches were needed: medication was prescribed that the patient did not use anymore (commission error) or patients used another drug at home (e.g. pantoprazole instead of omeprazole).

Upon discharge, start interventions were performed most frequently in the majority of the hospitals (e.g. restart of pre-admission medication that was temporarily discontinued).

#### Optimizations of medication

On admission, the proportion of patients with at least one optimization varied from 0 to 27% (0.4–3.7 interventions per patient) and upon discharge, this ranged from 2 to 95% of patients (0.1–1.7 interventions per patient). Highest numbers of optimizations were found in hospitals where the clarification step was integrated into the MedRec process, specifically on discontinuation of medication (e.g. discontinuing hypnotics that were initiated during admission and had no indication anymore upon discharge)(Fig. 2).

### Qualitative part

#### MedRec differences between hospitals

Analysis of the audio-transcript of the focus group emerged in three themes: *Who* performed MedRec, *Where* and *hoW* was MedRec performed. We combined and structured the three W’s with the results from the inventory questionnaires of participating hospital pharmacists and/or technicians (Table 2).

**Fig. 1:**
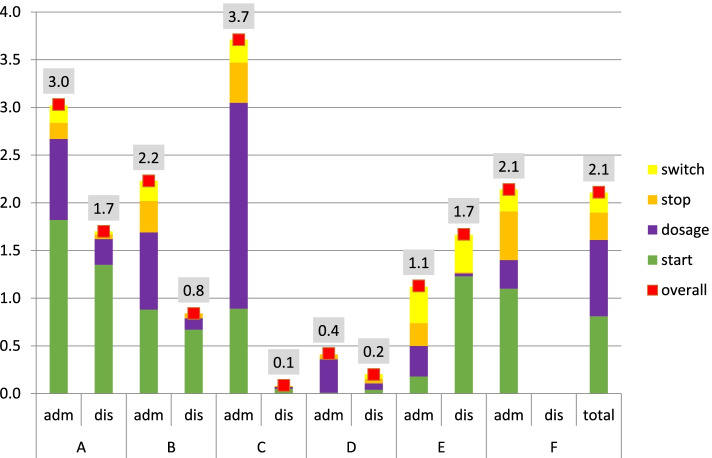
mean number and type of interventions per patient due to unintentional discrepancies upon admission/discharge

**Fig. 2:**
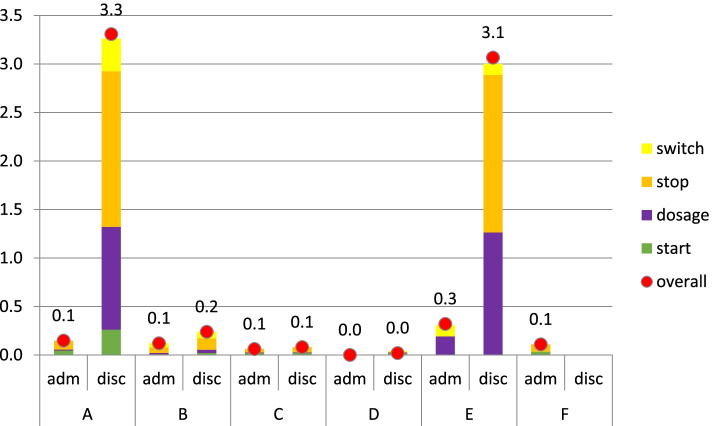
mean number and type of optimizations per patient per hospital on admission and discharge

*Who* ( interviewer, patient mix, physician type).

interviewer: highly trained MedRec interviewers resulted in high numbers of pharmacy interventions (see hospital A: having the highest numbers of interventions, and both higher educated and highly trained interviewers). All participants judged therapeutic knowledge of medicines as an important factor to apply optimization interventions (see below) in order to remove inappropriate and unnecessary medications from a patient’s medication list.

patient-mix/physician type: participants agreed that surgical patients generally have less discrepancies versus general wards as they use less medication. Also, participants reflected that a high socioeconomic status would result in less discrepancies due to the higher education level. This could explain the high number of interventions in hospital A with 75% of patients having a low socioeconomic status. Furthermore, all these patients were admitted at the pulmonary ward. In contrast: hospital B, C and D included a substantial percentage of surgical patients with the lowest number of interventions upon discharge (table 1, figures 1,2). According to participating pharmacists, surgeons generally will not act upon optimization interventions.

Hospital A and E had very comparable workflows but had different numbers of interventions on discrepancies: 3.0 versus 1.1 on admission (figure 1). Included patients differed highly: lung diseases only (hospital A), as compared to 70% internal medicine patients (hospital E, table 1). In participants‘ opinion, internal medicine doctors pay more attention to medication, probably resulting in a lower number of discrepancies (less found by the pharmacy team), even though high numbers of medication were used.

*Where* (at home, outpatient pharmacy or on the ward).

Pre-admission preparation at home: a form filled out by the patient himself (instead of using the CP medication history to perform MedRec) several weeks before elective admission, resulted in a shift of intervention type from start to dosage (figure 1, hospital C and D). Patients appeared to recall which medicines were used, but failed to note or remember dose and/or strength of a medication. This resulted in a high number of dosage interventions in hospital C and D, as compared to omissions in all other hospitals.

Location of the interviews (ward versus outpatient pharmacy): two hospitals, B and D, discussed discharge medication and counselled patients in the outpatient pharmacy only, instead of ward-based counselling. Those hospitals had at least 50% less interventions on discrepancies as compared to hospitals with ward-based MedRec and/or telephone interviews. This might have been the result of a less intense connection with in-hospital activities, according to participants.

*How* (optimizations and documentation).

Optimizations: hospitals differed in numbers and types of medication optimizations based on whether they embedded the optimization step or not. Hospitals that implemented optimization structurally included up to 8 focus-points on a checklist. The extensiveness of the checklist resulted in large differences in numbers of optimization interventions (figure 2). Furthermore, in hospital E, two pharmacists performed a medication review for selected patients (elderly, more medications) which potentially contributed to the higher number of optimization interventions, specifically upon discharge. This higher yield upon hospital discharge was explained by acceptance of certain clinical situations (in the context of medication use) while being admitted (e.g. accepting potassium suppletion in combination with potassium sparing medications while being admitted, but not upon hospital discharge without frequent laboratory control).

## Discussion

### Key findings

In this real-world setting study, quantitative and qualitative comparison of MedRec processes in 6 different hospitals revealed considerable differences in numbers of interventions: upon admission, patients with at least one discrepancy varied from 36–95%, while upon discharge, these numbers ranged from 5–28%.Optimizations reached 2% (admission) to 95% (discharge) of patients. Based on the qualitative analysis, we identified three main themes explaining differences between hospitals: patient- and healthcare professionals involved, where the patient interview was started or performed and to what extent medication optimization was embedded in the process (e.g. using checklists or per forming medication reviews).

### Comparison with previous work

In a published study (among 19 hospitals) the large variability between hospitals on performance of all steps was confirmed [28]. In this study, the variation was explained by obstacles like resource shortage, available staff and enthusiasm of the management. Comparable results were found in a study of the MedRec process in four acute care hospitals: varying levels of compliance with guidelines were noticed e.g. interview with the patient occurred in less than half of all MedRec (45.7%) [29]. Moreover, a very recent study revealed no implementation of MedRec in any of the observed sites [30]. Important barriers for MedRec implementation mentioned were: lack of awareness, a designated MedRec team and insufficient knowledge of health care professionals [14]. Apart from aforementioned obstacles and high risk patient population (elderly with or without polypharmacy), other factors like socioeconomic status and process-related factors have not been mentioned or investigated in previous studies. Even the recently published qualitative study among several US based hospitals was not conclusive on how MedRec should be implemented [31]. Therefore, our results give new insight in facilitators and barriers to perform MedRec activities within hospitals.

### Implications for practice

Our retrospective cohort without robust analysis should be interpreted with caution prior to further research. However, some specific topics may have the potential of a quick win in daily practice: differences in types of interventions were noticed amongst electively admitted patients having had the ability to fill out their medication list before admission. In that case, the majority of MedRec interventions were on dosage adaptions, whereas in situations lacking this patient list, medication was mostly started due to MedRec. Hence, the most common intervention type changed from omission to dosage, which might decrease the severity of the error from moderate to mild [32]. Furthermore, an optimization checklist increased the number of interventions on general wards. This effect on inappropriate medication use by standardization of a medication chart, especially with a high number of focus points, has been proven previously [33, 34]. However, no such effect was noticed on surgical wards in our study. This was explained by the fact that surgeons generally will not act upon optimization interventions and the high number of general, non-surgical focus points in the checklist.

Introduction of medication review (for selected patients) increased the intervention numbers as compared to the other hospitals (Fig. 1), giving room to a further improvement of health problems in daily live [35].

Upon discharge, some hospitals had very low numbers of interventions. Two potential explanations were given:1. high numbers of surgical patients in combination with 2. MedRec performed solely in the outpatient pharmacy (plus discharge medication dispensing). Probably, to prevent a surgical patient from medication related problems, either an admission interview only might be sufficient, (as reflected by the high number of discrepancy interventions on admission in hospital C) and/or a closer connection with the clinic (both pharmacy teams and physicians) may result in better communication and acceptance of more interventions.

An important implication for practice is that hospitals should determine what the achievement of MedRec should be. For example, many hospitals in our study focused on reducing discrepancies. However, this has no impact on sub- optimal pharmacotherapy like reducing unnecessary medication prescriptions upon hospital discharge (e.g. hypnotics, proton pump inhibitors, opioids). If the focus is also on optimizations, highly educated and trained staff may be needed.

### Strengths and limitations

To our knowledge this is the first multi-center analysis on MedRec processes providing insight into differences in interventions on medication discrepancies and—optimizations. Inclusion of a large and varied population (e.g. different patient categories and physician-types) increased generalizability and transferability; the connection of quantitative and qualitative results created the ability to improve our understanding of MedRec and give input for new research.

However, several limitations have to be mentioned. First, this study was performed in the Netherlands, reducing its external generalisability due to differences in health care systems. However, we expect that the main drivers between differences in MedRec interventions will not extremely differ between countries. Not all possible factors influencing the extent of MedRec are measured in the quantitative part of this study (e.g. specific patient characteristics like disease burden and workload of those who perform MedRec). Yet, the qualitative synthesis gave important insight and augmented also new facilitators and barriers for MedRec. This qualitative analysis though, could have been strengthened if pharmacy technicians and physicians had also been included in the focus group.

Third, documentation between hospitals differed and the retrospective nature of the study gave rise to a reporting bias as interventions were not always clear. This could result in an underestimation of the total number of interventions. However, this was not very frequent except for hospital F where we excluded the discharge results. Fourth, the actual impact of prevention of medication discrepancies on the individual patient has not been analysed. Therefore, we do not know if these discrepancies would have resulted in patient harm. However, Mekonnen et al. showed that a pharmacist-led MedRec program upon hospital transitions decreased ADE-related hospital revisits, all-cause readmissions and ED visits, resulting in a positive impact of MedRec [36].

## Conclusion

Hospitals differed greatly in the number of interventions performed during MedRec. Upon admission a variation of 0.4–3.7 interventions per patient was noted. Upon discharge the variation was 0.1–1.7 interventions per patient. A combination of patientmix, healthcare professionals involved, location and moment of the interview plus embedding and extent of medication optimization resulted in the highest yield of MedRec interventions.

## Supplementary Information

**Additional file 1: Box 1a.** Medication reconciliation process. **Box 1b.** Classification discrepancies and optimizations.

## Data Availability

All data generated during and/or analysed during the current study is available and included in this published article and its supplementary information files. If someone wants to request any further information about data and materials, CCM Stuijt can be contacted: stuijt@apomed.nl.
